# Analysis of the genetic diversity of *Candida* isolates obtained from diabetic patients and kidney transplant recipients

**DOI:** 10.1590/0074-02760160042

**Published:** 2016-06-07

**Authors:** Volmir Pitt Benedetti, Daiani Cristina Savi, Rodrigo Aluizio, Douglas Adamoski, Vanessa Kava-Cordeiro, Lygia V Galli-Terasawa, Chirlei Glienke

**Affiliations:** 1 Universidade Paranaense, Departamento de Microbiologia, Francisco Beltrão, PR, Brasil; 2 Universidade Federal do Paraná, Departamento de Patologia Básica, Curitiba, PR, Brasil; 3 Universidade Federal do Paraná, Departamento de Genética, Curitiba, PR, Brasil

**Keywords:** ITS1-5.8S-ITS2 and 28S rDNA, Candida, diabetes, renal transplant recipient

## Abstract

Yeasts of the genus *Candida* have high genetic variability and are the most common opportunistic pathogenic fungi in humans. In this study, we evaluated the genetic diversity among 120 isolates of *Candida* spp. obtained from diabetic patients, kidney transplant recipients and patients without any immune deficiencies from Paraná state, Brazil. The analysis was performed using the ITS1-5.8S-ITS2 region and a partial sequence of 28S rDNA. In the phylogenetic analysis, we observed a consistent separation of the species *C. albicans*, *C. dubliniensis*, *C. glabrata*, *C. tropicalis*, *C. parapsilosis*, *C. metapsilosis* and *C. orthopsilosis*, however with low intraspecific variability. In the analysis of the *C. albicans* species, two clades were formed. Clade A included the largest number of isolates (91.2%) and the majority of isolates from GenBank (71.4%). The phylogenetic analysis showed low intraspecific genetic diversity, and the genetic polymorphisms between *C. albicans* isolates were similar to genetic divergence found in other studies performed with isolates from Brazil. This low genetic diversity of isolates can be explained by the geographic proximity of the patients evaluated. It was observed that yeast colonisation was highest in renal transplant recipients and diabetic patients and that *C. albicans* was the species most frequently isolated.

Infections caused by opportunistic fungi, such as *Candida* yeast, often affect patients undergoing organ or bone marrow transplants, AIDS patients, patients taking immunosuppressive drugs, patients undergoing cancer treatment, those having undergone major surgery, those of advanced age or premature infants (Wisplinghoff et al. 2004). It can also affect individuals suffering from chronic stress, patients with metabolic diseases such as diabetes, those who are malnourished and those taking broad-spectrum antibiotics (Roden et al. 2005).

The majority of fungal infections in humans are caused by the species *C. albicans* and *C. glabrata*. The prevalence rates of *C. albicans* and *C. glabrata* infections are approximately 70% and 15%, respectively (Kolaczkowski et al. 2010). Infections caused by non-*albicans Candida* (NAC) species, such as *C. tropicalis*, *C. parapsilosis*, *C. krusei*, *C. lusitaniae*, *C. inconspicua*, *C. lipolytica* and *C. norvegensis*, have become increasingly more frequent; in some cases, infections with NAC species are predominant (Pfaller & Diekema 2007, Kothavade et al. 2010). There is great genetic diversity among different yeast species, in particular *C. albicans*, and this characteristic may be explained by the presence of a diploid genome, predominantly clonal reproduction and a high rate of recombination (Jacobsen et al. 2008).

In the present study, we evaluated the genetic diversity among *C. albicans* isolates from diabetic patients and kidney transplant recipients and compared them to other isolates described in the literature. We also determined which species of *Candida* were involved in the colonisation of the oral cavity in diabetic patients and renal transplant recipients from southern Paraná state (Brazil), including with what frequency colonisation occurred. We also evaluated the intraspecific diversity of *C. albicans* and its population structure.

## MATERIALS AND METHODS

*Patients analysed* - In total, 190 individuals were analysed, of which 64 were diabetic patients, 37 were kidney transplant recipients, and 89 had no immune deficiencies (control group). The diabetic patients were over 40 years old, had been diagnosed with type II diabetes for over five years, were not using insulin and had hypertension; 48 had hyperglycaemia. All transplant patients were over 30 years old and had a kidney transplant over one year ago; 19 patients were on the immunosuppressant prednisone. The control group was composed of people who were between the ages of 18 and 30, were not being treated for any disease and were not using drugs with antimicrobial or anti-inflammatory activities. An epidemiological survey of the patients was also performed to obtain more information.

*Sample processing* - Approximately 1 mL of saliva was collected from each patient according to the no stimulation method described by Navazesh & Kumar (2008). After collection, 100 mL of saliva was inoculated in CHROagar® medium (Becton-Dickinson, Franklin Lakes, New Jersey, USA) and incubated at 25ºC for five days. After incubation, the colony-forming units per mL saliva (CFU/mL) were determined. An initial screening of *Candida* was performed to assess biochemical assimilation (auxonograma), sugar fermentation (zymogram) and production of germ tubes (Kurtzman & Fell 1998). Isolates were maintained by inoculating in Brain Heart Infusion medium (Difco) containing 20% glycerol in Eppendorf tubes and storing at -20ºC (Silva et al. 2008).

*Isolates and reference strains* - The present study examined 120 yeast species isolated from 96 patients out of a 190-patient pool. The following reference *Candida* strains from the American Type Culture Collection (ATCC) were also used: *C. albicans* ATCC 44858, *C. glabrata* ATCC 2001 and *C. tropicalis* ATCC 28707. This research was approved by the Ethics Committee under registration number CAAE-0200.1.375.000-11 - Paranaense University, Paraná (PR), Brazil).

*DNA extraction* - Genomic DNA was extracted using an Ultraclean Microbial DNA Isolation Kit (MoBio^®^) according to the manufacturer’s instructions and stored at -20ºC after extraction.

*Amplification of the ITS1, 5.8S, ITS2 and 28S rDNA regions* - The primers V9G (de Hoog & van den Ende 1998) and ITS4 (White & Morrow 1990) were used to amplify the Internal Transcribed Spacer (ITS) regions and 5.8S rDNA. The primers LR0R and LR5 were used to amplify fragments of 28S rDNA (Vilgalys & Hester 1990). Polymerase chain reaction (PCR) reactions were performed in a total volume of 25 μL, which contained Tris Base buffer solution (pH 8.4) (20 mM), KCl (50 mM), deoxynucleotide triphosphates (dNTPs) (0.3 mM) (Invitrogen-Life Technologies, Brazil), MgCl_2_ (1.6 mM), primers (15 pmol each), Taq DNA polymerase (0.5 U) (Invitrogen-Life Technologies, Brazil) and template DNA (20 ng). The amplification of the ITS regions and the 5.8S gene was performed using the following protocol: 95ºC for 5 min; 30 cycles of 95ºC for 1 min, 57ºC for 1 min, and 72ºC for 1 min; and a final step at 72ºC for 5 min. The amplification of the 28S region was performed according to the following protocol: 95ºC for 5 min; 30 cycles at 95ºC for 1 min, 48ºC for 1 min, and 72ºC for 1 min; and a final step at 72ºC for 5 min.

*PCR product purification* - The PCR products (25 µL) were purified using 7.5 M ammonium acetate (15 µL) and absolute ethanol (74 µL). Samples were incubated on ice for 1 h, followed by centrifugation for 45 min at 23,100 g. The pellet was suspended in 12 µL of MilliQ water.

*rDNA sequencing* - Sequencing of the PCR products was performed using an ET Kit (DYEnamic ET Dye Terminator Cycle Sequencing for MegaBACE - Amersham Biosciences^®^) according to the manufacturer’s instructions. The products of the sequencing reaction were purified using Sephadex^™^ G-50 Fine DNA Grade resin and subjected to analysis by electrophoresis in a MegaBACE (Amersham Biosciences^®^) automated DNA sequencer.

*Phylogenetic analysis* - BioEdit 7.1.9 (Hall 1999) and MEGA 5.1 (Kumar et al. 2008) software were used for sequence editing and alignment. GARLI (Zwickl 2006) software was used for the maximum likelihood phylogenetic analysis, and MrBayes v.3.2.1 (Ronquist & Huelsenbeck 2003) software was used for Bayesian inference analysis. A bootstrap procedure with 2,000 replicates was used to check node consistency. In the analyses of maximum likelihood and Bayesian inference, ModelTest software version 3.7 (Posada & Crandall 1998) was used to create the evolutionary model. A phylogenetic tree was generated using sequences from 80 isolates, the ATCC - 44858 (KM361826) reference lineage, lineages from Paraná state [UFPR - HC04IC (KJ651886)] and from São Paulo state (USP - ICB945 (JX463265), UFC - CA1150 (AB861482), UNICAMP - CA70 (DQ141236), UNESP - CA15 (KF385990) and the UNIFESP isolates LEMI7986E (KC905077) and L8278 (KC408953). The sequence from the type strain CBS - 562 (NR125332) of *C. albicans* was also included in the analysis. All of the sequences analysed in this study were deposited in GenBank (KM361747- KM361866 and KM464557- KM464676) (http://www.ncbi.nlm.nih.gov/genbank/).

## RESULTS

*Epidemiological aspects of isolated yeast* - Of the 190 patient samples, 96 (50.53%) contained yeast growth. A total of 120 strains belonging to the genus *Candida* were isolated from these 96 patient samples*.* Among the 37 samples from transplant recipients, yeast growth was observed in 19 (51.35%); the average CFU/mL of the saliva was 814. Among the 64 samples analysed from patients with diabetes, 44 (68.75%) contained yeast growth; the average CFU/mL was 932. In the control group (89 patients), growth was observed in 33 (37.08%) samples; the average CFU/mL of the saliva was 215 (Table). Of the 120 isolates, 80 strains were identified as *C. albicans*, 17 as *C. parapsilosis*, eight as *C. tropicalis*, six as *C. glabrata*, four as *C. dubliniensis*, three as *C. metapsilosis* and two as *C. orthopsilosis* (Table). The highest average CFU/mL was observed in patients with diabetes and kidney transplants relative to the control group (p = 0.01). Average CFU/mL values were 879 for *C. parapsilosis*, 854 for *C. tropicalis*, 649 for *C. albicans*, 520 for *C. glabrata*, 55 for *C. dubliniensis*, 47 for *C. metapsilosis* and 25 for *C. orthopsilosis*. The analysis of patient interview data revealed that gender did not affect yeast growth, as the growth index was 24 (53.3%) among men and 72 (49.7%) among women (p = 0.90). There was also no correlation found among the different age groups (< 50 years, 51-60 years and > 60 years) in terms of yeast isolation (p = 0.09). Past candidiasis did not influence colonisation because yeast was isolated from 44 (45.8%) patients with a previous history of oral candidiasis and from 52 (54.2%) of patients with no history of oral candidiasis (p = 0.13).

**TABLE:** Epidemiological data of patients from whom yeast isolates were collected

Patients	Epidemiological characteristics		

	Isolation frequency ^a^(n)	Average CFU/mL^b^	Species isolated ^c^(n)
Kidney transplant	51.35% (19)	814	*Candida albicans* (15) *C. parapsilosis* (1) *C. glabrata* (2) *C. tropicalis* (2) *C. metapsilosis* (1) *C. orthopsilosis* (1)
Diabetic	68.75% (44)	932	*C. albicans* (39) *C. parapsilosis* (10) *C. glabrata* (1) *C. tropicalis* (5) *C. dubliniensis* (1) *C. metapsilosis* (2) *C. orthopsilosis* (1)
Control group	37.08% (33)	215	*C. albicans* (26) *C. parapsilosis* (6) *C. glabrata* (3) *C. tropicalis* (1) *C. dubliniensis* (3)

a: number of patients whose saliva samples contained yeast.

b: CFU/mL - colony-forming units per 1 mL of saliva.

c: number of *Candida* species isolated per patient type.

**Fig. 1:**
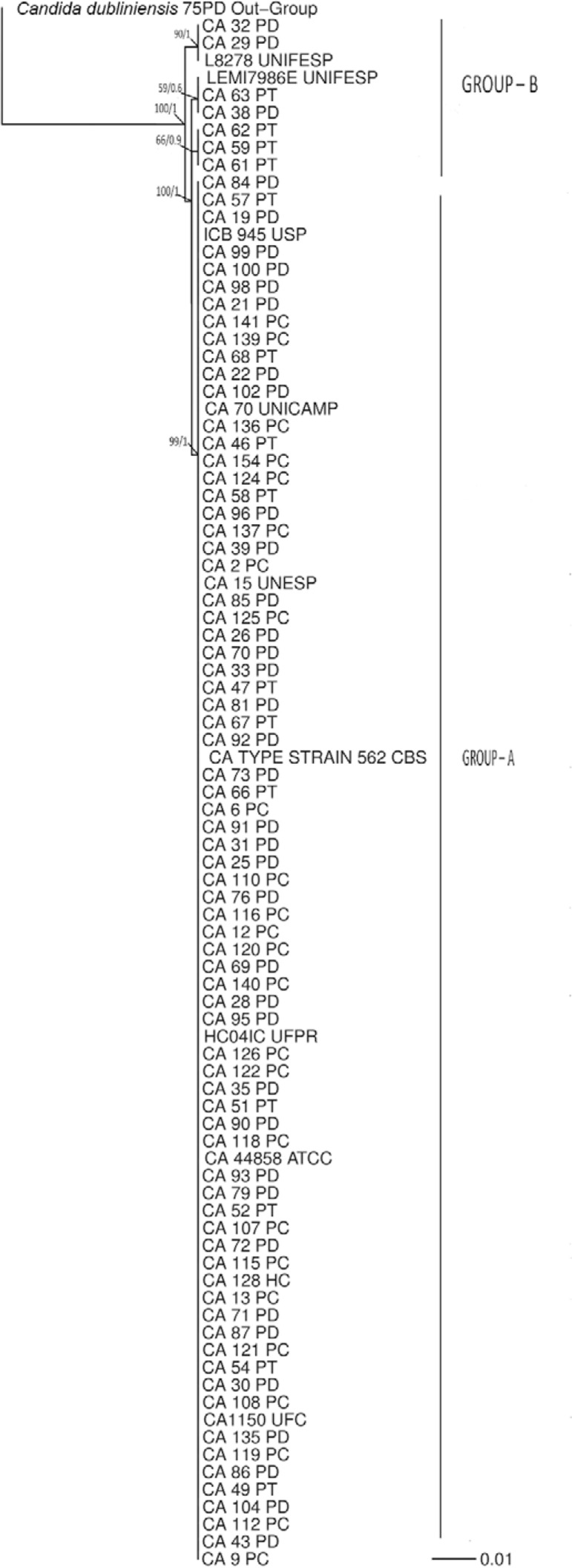
: Bayesian inference phylogenetic tree of *Candida albicans* isolates and reference strains generated using ITS1-5.8S-ITS2 of rDNA sequences. Bootstrap values of maximum likelihood and Bayesian posterior probability values are shown above nodes. The *C. dubliniensis* isolate was used as the outgroup. The scale bar indicates the number of expected changes per site.

*Phylogenetic analysis* - To determine the intraspecific variability among *C. albicans* isolates, our isolates were compared to sequences of *C. albicans* isolated from Brazil that were deposited in GenBank. In the ITS1-5.8S-ITS2 phylogenetic tree of *C. albicans* isolates (Fig. 1), we observed the possible formation of two population groups. Clade A consists of the largest number of isolates, including ATCC-44858, the type strain *C. albicans* - CBS562 and isolates from different Brazilian states including Paraná state (HC04IC/UFPR, KJ651886/GB), São Paulo state (ICB945/USP, JX463265/GB; CA70/UNICAMP, DQ141236/GB; and CA15/UNESP, KF385990/GB) and Ceará state (CA1150/UFC, AB861482/GB) (Neto et al. 2014). Isolates belonging to Clade A had no genetic divergence in the ITS1-5.8S-ITS2 rDNA region. The isolates with the highest genetic diversity were in Clade B (CA29PD, CA32PD, CA38PD, CA59PT, CA61PT, CA62PT, CA63PT and CA84PD) (Fig. 1).

**Fig. 2:**
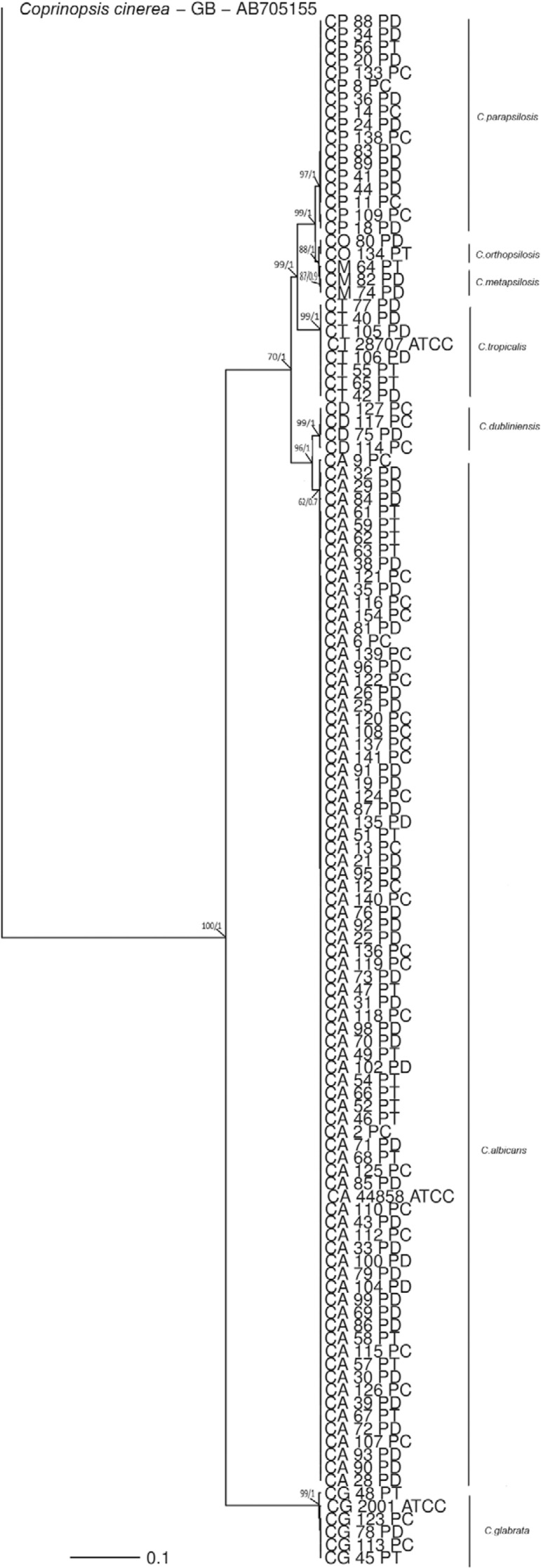
: Bayesian inference phylogenetic tree of seven *Candida* species. The tree was built using concatenated sequences of the ITS and 28S genes. Bootstrap values of maximum likelihood and Bayesian posterior probability values are shown above nodes. The *Coprinopsis cinerea* isolate was used as the outgroup. The scale bar indicates the number of expected changes per site.

In the phylogenetic tree, assembled with 120 isolates belonging to seven different *Candida* species in which sequences from the ITS1 and ITS2 regions and the 5.8S and 28S rDNA genes were used, there is a consistent separation of the following species: *C. albicans*, *C. dubliniensis*, *C. glabrata*, *C. tropicalis* and the three species that formed the *C. parapsilosis* complex (*C. parapsilosis*, *C. metapsilosis* and *C. orthopsilosis*). Moreover, we were able to observe low intraspecific variability (Fig. 2).

## DISCUSSION

Analyses of different epidemiological aspects of the *Candida* species may assist in disease prevention, control and treatment. In our analysis, yeast colonisation was more frequently observed in renal transplant recipients and diabetic patients (Table) than in the control group. This finding indicates that patients with altered immune responses are more susceptible to fungal infection, which is in agreement with Kothavade et al. (2010). The high *Candida* colonisation observed in diabetic patients is correlated with multifactorial events that are influenced by factors such as high concentrations of sugars (sucrose, glucose and fructose) in tissues and low levels of salivary secretions (Khovidhunkit et al. 2009). A high frequency of colonisation in diabetic patients has been previously observed (Menezes et al. 2007, Sashikumar & Kannan 2010, Obradović et al. 2011). In addition, Colombo et al. (2012) reported that diabetes mellitus is one of the most common pathological disorders predisposing adult patients to the development of different clinical forms of candidiasis.

Our results show that *C. albicans* was the most frequently isolated species among all patient groups (Table). *C. albicans* is a yeast species of wide genetic variability and great adaptability to different anatomical sites, likely explaining its high frequency of isolation from human hosts (Zhou & Lorenz 2008, Yan et al. 2009). A clear example of this correlation between colonisation and pathogenicity is the fact that *C. albicans* is responsible for 90% of oral and oesophageal candidiasis episodes (Colombo et al. 2012).

Although *C. albicans* is still the most common pathogen involved in candidiasis, a significant increase in NAC infections has been observed recently (Arancia et al. 2009); in particular, *C. glabrata*, *C. krusei*, *C. tropicalis* and *C. parapsilosis* have been found to be etiological agents of disease (Silva 2014). In our study, one-third of the isolates identified were NAC species (Table). According to Hsueh et al. (2005), increases in colonisation with NAC species are associated with extensive prophylactic use of antifungal drugs in immunocompromised patients. It was also observed that *C. parapsilosis* was frequently isolated as an accompanying species in the present study. This result corroborates the guidelines for the management of candidiasis as described by Colombo et al. (2012). Here, the authors classified *C. parapsilosis* as one of the most common agents of candidaemia in Latin America, in particular Brazil. This type of infection mainly affects hospitalised patients using central venous catheters.

In our study, certain epidemiological aspects, such as gender, age and candidiasis history, did not influence the colonisation of *Candida* species in the oral cavity of the analysed patients. These results confirm those of Obradović et al. (2011), who determined that oral cavity colonisation was not associated with gender. Our results are also in agreement with studies by Belazi et al. (2005), who did not observe an association between oral candidiasis and patient gender or age. Hof (2010) suggests that increases in the frequency of oral candidiasis may be associated with poor oral hygiene, specific diets, use of dental prosthesis or other diseases of the oral mucosa.

The genetic diversity among *C. albicans* isolates was analysed (Fig. 1), and the formation of two population groups was observed. Group A consisted of the largest number of isolates in this study, as well the type strain and strains from other Brazilian states. Group B included seven isolates and the strains LEMI7986E (KC905077/GB) and L8278 (KC408953/GB) belonging to UNIFESP University (São Paulo state) (Merseguel et al. 2015). Despite the formation of two groups, low genetic diversity was observed between the isolates collected from Paraná state (Federal University of Paraná) when compared to strains from different Brazilian states. It was also observed that specific phylogenetic groups were not formed based on the type of patient (renal transplant recipient, diabetic or control group). The two groups formed were 98.8% similar with five polymorphic sites in the 412 bp analysed (four sites in the ITS region and one site in the 5.8S region). However, this last substitution is a mutation. Therefore, we suggest that regions other than ITS and 5.8S should be analysed for intraspecific studies of this group.

Some studies have suggested that species of *C. albicans*, although isolated from different patients, can have low variability despite having very similar genomes (Odds et al. 2006). *Candida* spp, which is present in many different anatomical sites in the human body, may have passed from one individual to another as populations migrated; this would explain the low genomic variability observed (Odds et al. 2008). Additionally, some studies suggest that most individuals are colonised or infected by one single strain of *Candida*; more significant genetic differences may exist between isolates of different geographic origins (Soll & Pujol 2003). Large diversity in a population of *C. albicans* may be the result of different sources, as observed in the work of Shin et al. (2011) and Hammarskjöld et al. (2013). The low variability found among the *C. albicans* isolates in the present study could be explained by the geographic proximity of the patients, as was determined in studies by Martens et al. (2007) and Aserse et al. (2012).

A phylogenetic tree was formed using the sequences of seven different *Candida* species (Fig. 2) and showed the formation of distinct branches for each species. However, with low intraspecific variability and despite the genetic proximity of the *C. parapsilosis* complex (Roy & Meyer 1998), the separation of the complex into distinct species was required, as was also reported by Tavanti et al. (2005) and Carvalho et al. (2013).

In conclusion, yeast colonisation of the oral cavity occurs more frequently in renal transplant recipients and diabetic patients than in healthy individuals. Sequences of ITS and rDNA regions were able to correctly identify *Candida* species and can be used for accurate species identification for this genus. *C. albicans* was the most frequently isolated species regardless of patient gender or age. The phylogenetic study showed that *C. albicans* isolates have low genetic diversity based on rDNA sequences, and the formation of two population groups was observed. However, these groups had no correlation with respect to gender, age or region of isolation.
